# Choline and betaine concentrations in plasma discriminate levels of dietary choline intake in healthy adults: analysis of a double-blind randomized crossover controlled feeding study

**DOI:** 10.1016/j.ajcnut.2026.101236

**Published:** 2026-02-11

**Authors:** Isis Trujillo-Gonzalez, David A Horita, Julie Stegall, Rachel Coble, Evan M Paules, Anju A Lulla, Emmanuel Baah, Teodoro Bottiglieri, Wei Sha, Martin Kohlmeier, Walter B Friday, Steven H Zeisel

**Affiliations:** 1 Nutrition Research Institute, University of North Carolina at Chapel Hill, Kannapolis, NC, United States; 2 Department of Nutrition, Gillings School of Global Public Health, University of North Carolina at Chapel Hill, Chapel Hill, NC, United States; 3 Center of Metabolomics, Institute of Metabolic Disease, Baylor Scott & White Research Institute, Dallas, TX, United States; 4 Department of Cancer Biostatistics, Levine Cancer Institute, Atrium Health, Charlotte, NC, United States

**Keywords:** dietary intake biomarkers, choline, betaine, fatty liver, stable isotope tracer

## Abstract

**Background:**

Choline is an essential nutrient, and insufficient intake negatively affects the liver, brain, and muscles. In the United States, habitual choline intake remains below the adequate intake (AI). To date, no circulating metabolites have been validated to distinguish between low and adequate choline intake.

**Objectives:**

We tested whether plasma concentrations of choline and its metabolites could discriminate adequate compared with low dietary choline intake and whether liver elastography (FibroScan) could detect diet-induced changes in liver fat.

**Methods:**

In a double-blind, randomized, crossover feeding study, participants followed three 15-d dietary arms providing ~100%, 50%, and 25% of the choline AI in the form of choline chloride. On day 12 of each dietary arm, participants consumed a single bolus of 2.2 mmol trimethyl-d_9_-choline. Targeted assays quantified plasma choline, betaine, phosphatidylcholine (PtdCho), and total homocysteine (tHcy) concentrations. Liver fat content was measured using FibroScan.

**Results:**

Plasma concentrations of d_9_-choline, betaine, and their isotopic enrichment ratio (IER) varied with dietary intake (*q* < 0.0001), and PtdCho IER also differed significantly (*q* = 0.001). In targeted analysis, choline and betaine concentrations were highly responsive to dietary choline intake, whereas PtdCho and tHcy were not. Compared with the 100% AI arm, plasma choline was lower in the 25% AI arm [β= −2.20, 95% confidence interval (CI): −2.72, −1.68]. Receiver operator characteristic analysis showed strong discrimination for plasma choline [area under the curve (AUC) = 0.81, 95% CI: 0.74, 0.88], and betaine (AUC = 0.80, 95% CI: 0.73, 0.88), with improved discrimination when combined (AUC = 0.85, 95% CI: 0.79, 0.91). Fibroscan identified a subset of participants with increased liver fat in response to the 25% AI compared with 100% AI choline diet, though patterns varied among individuals.

**Conclusions:**

Plasma choline and betaine concentrations discriminate low compared with AI under controlled feeding. These findings support targeted metabolite profiling to improve choline intake assessment and reveal individual differences in liver response to low choline intake.

This study was registered at Choline Nutritional Status: Development of a Biomarker Panel as NCT03726671 (www.clinicaltrials.gov) registered 31 October, 2018.

## Introduction

Choline is an essential nutrient that serves as a precursor for several metabolites critical for human health. These metabolites include phosphatidylcholine (PtdCho), a major structural component of cell membranes; acetylcholine, a neurotransmitter; and betaine, a methyl donor involved in the regulation of gene expression [1,2]. Although choline can be synthesized de novo from phosphatidylethanolamine via the enzyme phosphatidylethanolamine-*N*-methyltransferase (PEMT), endogenous synthesis alone is insufficient to meet daily choline requirements for most people. Therefore, dietary intake of choline is necessary to maintain optimal health. Low dietary choline intake in adults is associated with adverse effects such as hepatic fat accumulation and muscle dysfunction [3,4], and underconsumption of choline during pregnancy increases the risk of having a baby with neural tube defects [5].

The adequate intake (AI) levels for choline were established by the Institute of Medicine (now the National Academy of Medicine) as part of the United States Dietary Reference Intakes in 1998 [6]. The United States reference values recommend 550 mg/d formales and 425 mg/d for females, increasing to 450 mg/d during pregnancy and lactation [2]. Data from the 2013–2016 NHANES survey in the United States indicate that most individuals do not meet these recommendations [7,8]. In addition, studies using food frequency questionnaires have highlighted a widespread trend of low choline dietary intake among females of reproductive age. For example, in the Gambia, intake in premenopausal females was estimated at just 155 mg/d [9]. In Mexico and Taiwan, reported intakes are ~262 and 284 mg/d, respectively [10,11]. However, the accuracy of these dietary assessments is limited by the inherent weaknesses of food frequency questionnaires, including imprecise questions, lack of detail on food items, and underreporting. These limitations raise concerns about the reliability of using such data to assess choline intake [12].

Genetic variation adds a layer of complexity to choline metabolism. Single-nucleotide polymorphisms (SNPs), such as rs12325817, located in the promoter region of the *PEMT* gene, can reduce gene expression and thereby increase reliance on dietary choline [13]. Moreover, the promoter region of the *PEMT* gene is regulated by estrogen-responsive elements [14,15], which partly explains why premenopausal females consuming a low choline diet are less likely to experience organ dysfunctions associated with low choline intake compared with menopausal females andmales [16]. In controlled feeding studies, choline deprivation in males led to fatty liver, elevated plasma levels of hepatic enzymes such as aspartate aminotransferase (AST) and alanine aminotransferase (ALT), and increased muscle breakdown indicated by elevated plasma creatine kinase (CK) [4,17]. Although choline is recognized as an essential nutrient and recent advances have shed light on choline transport [18, 19], our ability to accurately measure dietary choline intake in human populations remains limited.

To address this gap, we focused on identifying plasma metabolites that can discriminate between adequate and low choline intake. Our study represents an intermediate step toward developing a validated dietary biomarker, which must demonstrate a dose-dependent response to intake and external validity across populations [20]. In this study, we tested whether plasma metabolites can reliably indicate low choline intake under controlled feeding conditions. Given the central role of choline and its metabolites in maintaining metabolic and organ health, this study evaluates approaches to estimate dietary choline intake before clinical manifestations occur, identifying plasma metabolites as the most informative indicators of low choline.

## Methods

### Study design

The study design was a longitudinal, double-blind, randomized crossover dietary intervention (clinicaltrials.gov identifier: NCT03726671). Recruitment and study activities occurred between 1 November, 2018 and 20 October, 2021. Choline content of the dietary intervention was based on the National Institute of Medicine-defined AI for adult males (550 mg choline/d) [21]. Participants were provided with all foods, drinks, and snacks for the duration of each of the three 15 d controlled feeding arms followed by a 2-wk washout period in which the participants returned to their usual diet. No additional follow-up was conducted after completion of the third arm. These diets were identical except for the modification of the amount of choline (as choline chloride), added to 3 bread rolls/d.

#### Choline roll preparation and diet composition

Choline content and stability during choline storage in the bread rolls were determined separately for each batch produced. Choline chloride and riboflavin were dissolved in water and incorporated into the dough mixture during roll preparation (21 g, 6.27 g, and 0 g of choline chloride per batch for 100%, 50%, and 25% AI rolls, respectively). Each batch produced 120 rolls, portioned by weight (±1%) to ensure consistent choline and riboflavin content across servings. Choline and betaine concentrations in homogenized composite samples of the rolls were verified by liquid chromatography (LC)-mass spectrometry (MS). Nutrient composition values for the full diets were taken by the commercial food supplier. The nutrient composition of the complete diets, including choline and betaine content, is presented in Supplementary Table 1, and the detailed ingredient composition and choline standardization of the bread rolls are provided in Supplementary Table 2.

#### Diet administration and monitoring

The amount of choline in each diet was not adjusted for subject sex or body mass index (BMI). Body weight was monitored at screening and on study days 1, 5, 12, and 15 of each dietary arm. No significant changes within or between arms were observed, confirming weight stability. Particular attention was given to participants near the lower and upper limits of the inclusionary BMI range (20–30 kg/m^2^). Interestingly, a few participants showed a mild tendency toward weight loss despite the controlled diet. These participants were counseled and provided with additional study-approved snacks (soda, pears, and rice; Supplementary Table 1) to maintain energy balance. Participants were instructed not to engage in additional exercise during this period. All study diets were designed to provide approximately equal amounts of betaine, averaging147 mg/d betaine. Adverse effects were actively monitored throughout all visits. Participants were asked about new symptoms at each encounter, and safety laboratory measures including ALT, AST, and CK were reviewed under physician oversight. No adverse events related to the intervention were observed.

#### Randomization

Study participants received 3 dietary arms in a randomized order generated using a computer-based randomization plan (www.randomization.com). Diet-order permutations were created, and participants were assigned sequentially by the study coordinator to the next available randomization slot. Allocation concealment was maintained through coded labeling (A, B, and C) of choline diets, known only to 1 unblinded staff member responsible for roll preparation. The rest of the study team remained blinded to arm assignment. The dietary arms delivered 5.1 mmol choline/d (531 mg, 97% AI), 2.5 mmol choline/d (47% AI), or 1.3 mmol choline/d (25% AI). A minimum of 2-wk washout period separated each arm, during which the participants returned to their usual diet.

#### Tracer administration

On day 12 of each dietary arm, in addition to the food supplied, subjects consumed a single bolus of 2.2 mmol trimethyl-d_9_-choline (as 98% D, methyl-perdeuterated chloride; Cambridge Isotope Laboratories). The selected dose was adapted from Pynn et al. [22], who used an intravenous infusion design, and was increased by ~25% to account for expected differences in absorption with oral delivery. This approach allowed us to achieve physiologically relevant enrichment levels while maintaining the dietary nature of the intervention. We confirmed total choline and betaine content of diets using duplicate food portions analyzed by LC-stable isotope dilution–multiple reaction monitoring (SID-MRM)/MS as previously described [23].

#### Compliance assessment

Riboflavin (30 mg/d) was added to the bread rolls and was used as a compliance biomarker via 24-h urine collection measures [24–26]. 24 h-urinary excretion of riboflavin was measured at baseline (day 1) and on day 15 of each arm. Compliance was assessed based on an increase in riboflavin excretion of ≥1 mg/mL over individual baselines [25]. Participants who were noncompliant in 2 arms were excluded from all the analyses. Although all participants completed the 3 study arms, compliance was assessed only after study completion based on riboflavin measurements. Three participants were excluded due to noncompliance (1 male, 1 premenopausal female, and 1 menopausal female), and 2 additional participants completed the 100% AI and the 25% AI choline diet arms but withdrew midway through the 50% AI arm.

#### Blood collection and sample size

Blood samples were collected by venipuncture on days 1, 12, 13, and 15. Sample size expectations were informed by an interim analysis from this study published in Horita et al. [27], which used an interim subset to evaluate isotope dilution MS of plasma and in vivo single-voxel liver magnetic resonance spectroscopy (MRS). Power calculations performed using G*Power (version 3.1.9.7) [28], under a repeated measures analysis of variance (ANOVA) structure (effect size *d* = 0.5, *P* = 0.05) indicated that a total sample of 30 participants would provide 80% power to detect diet effects. Our final analytic sample of 73–75 participants, therefore, exceeds the sample size demonstrated to provide adequate power for detecting diet-related differences.

#### Ethics

The protocol was approved by the Institutional Review Board at the University of North Carolina at Chapel Hill, and informed consent was obtained.

### Subject recruitment

#### Inclusion/exclusion criteria

All participants were recruited from the greater Kannapolis-Charlotte, NC metro area and screened by a physician and by self-report assessment. Participants self-identified their background from predefined categories on the screening form. The response options were: White, Black or African American, Hispanic/Latino, Asian, Native American/Alaska Native, Pacific Islander and “More than one race.” Participants could select >1 category. These descriptors were used only for sample characterization and not as explanatory variables in the analyses. Clinical laboratory tests were run, including a comprehensive metabolic panel. Menopausal status was classified using serum follicle-stimulating hormone (FSH) and estrogen concentrations measured by LabCorp.

Inclusion criteria were the age range of 20–67 y and BMI range of 20–30 kg/m^2^. Exclusion criteria included medication known to alter or damage liver or muscle health, and those that have the potential to alter choline metabolism. Participants with chronic systemic disease (including metabolic, hepatic, renal, cardiovascular, or gastrointestinal disorders such as Crohn’s disease, ulcerative colitis, and celiac disease), a history of hepatic or renal disease, being a substance abuser, current smoker, or alcohol consumption of >2 alcoholic beverages/d (14/wk) were also excluded, as were participants consuming choline-containing dietary supplements, frequently consuming a diet that might interfere with the study, or having allergies to soy proteins or any foods in the required diet. Females who were pregnant, breastfeeding, or planning to become pregnant were excluded due to the potential risk to the fetus/baby of a low choline diet. Subjects who performed intense exercise of more than 1 h every day or other intense muscle-building exercises (such as weightlifting low-weight maintenance repetitions) were also excluded.

**FIGURE 1.**
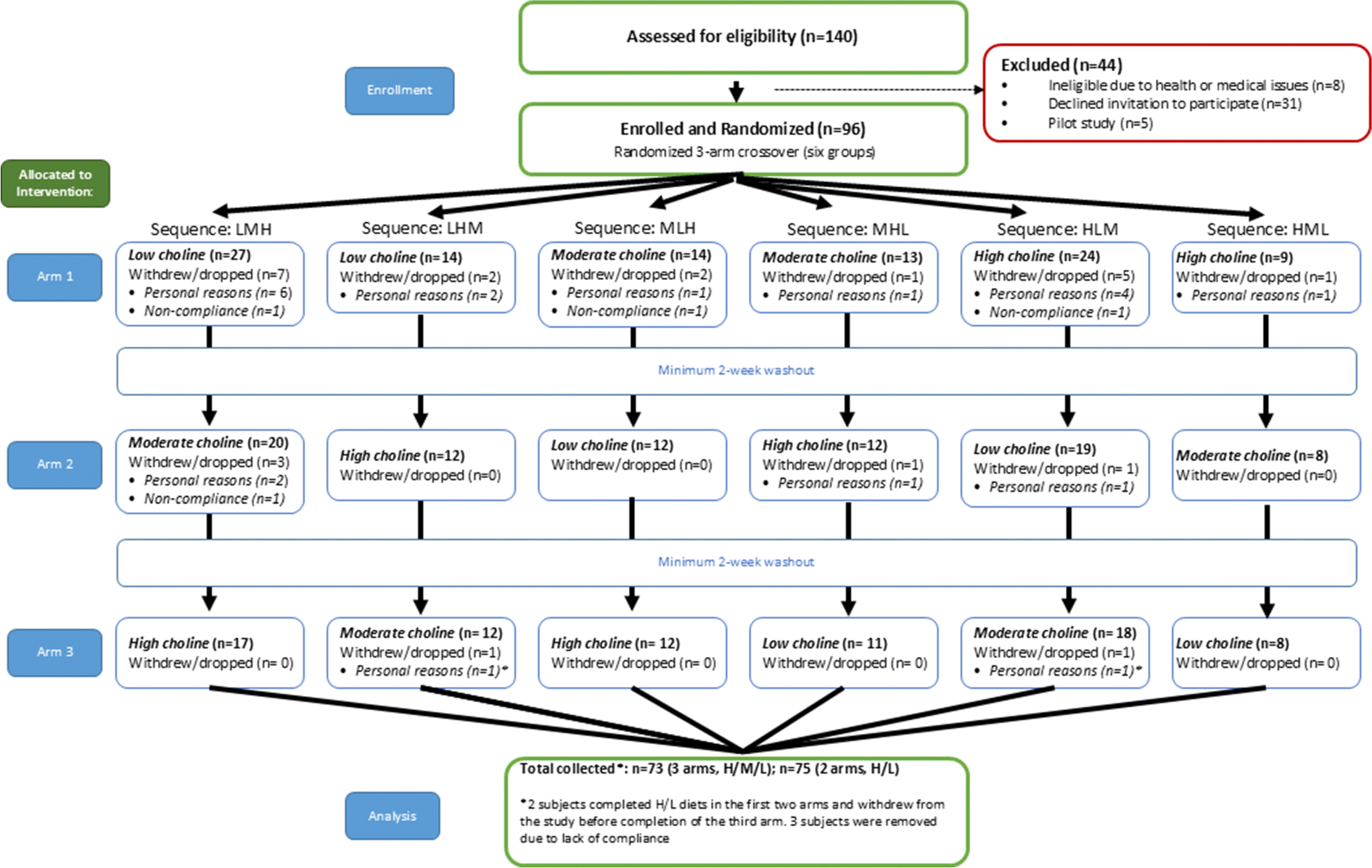
CONSORT diagram. Flowchart of participants through the study. Low choline 25% AI choline intake; Medium choline 50% AI choline intake; High choline, 100% AI choline intake. AI, adequate intake.

A total of 140 individuals were assessed for eligibility; 44 were excluded (31 declined participation, 8 did not meet the inclusion criteria or had medical issues, and 5 participated only in an early pilot phase). A total of 96 healthy volunteers were enrolled and randomly assigned into the controlled feeding trial (50 premenopausal females, 20 postmenopausal females, 1 perimenopausal female, and 30males) (Table 1). A flow chart diagram is included (Figure 1). Because participant recruitment occurred during the COVID-19 pandemic, study visits were suspended beginning in March 2022 and resumed in June 2023. COVID-19 symptoms and travel screenings were conducted within 24 h before each visit and again on arrival. Body temperature was checked at entry, and masks were required for both staff and participants throughout the visit, with additional eye protection used as needed. Social distancing was maintained by modifying procedures, for example, participants self-operated the body composition scale and stadiometer under staff supervision. All surfaces and equipment were sanitized before and after each visit. Two institutional review board-approved protocol amendments were submitted to implement and formalize these safety measures.

#### Sample collection

All subjects were seen at the Human Research Core at the University of North Carolina Chapel Hill, Nutrition Research Institute, Kannapolis, NC and fasted overnight before blood collection. Whole blood was collected in serum-separating tubes (SST) for hormone measurements and lithium heparin tubes for plasma metabolite analysis. Tubes were gently inverted to mix. Lithium heparin tubes were immediately put on wet ice and centrifuged at 2200 × *g* at 4°C for 10 min, then returned to the ice bath. Plasma samples were pooled, aliquoted, and frozen at −80°C within an hour of collection time. SST tubes were allowed to clot at room temperature, then centrifuged at 2200 × *g* at 4°C for 10 min. SST tubes were sent for a comprehensive metabolic panel and CK testing to the clinical laboratories at Lab Corporation of America, Burlington, NC, which is both Clinical Laboratory Improvement Act (CLIA ID 34D0655059) and College of American Pathologists (CAP No. 1396901) accredited. At enrollment, FSH and estrogen were also measured in female participants.

Urine was collected by participants into a commode specimen collector and immediately poured into a 3-L plastic jug that contained 3 mL acetic acid. The plastic jug was kept cool with ice packs in an insulated cooler during the 24-h collection. On completion, the total volume and collection times were noted. Aliquots of 1 mL were created with and without formic acid (10 μL) and stored at −80°C.

Tanita Body Composition Analyzer was used for weight and body composition measures following the manufacturer’s instructions. Participants were weighed fully clothed but with shoes and socks removed. Height used for entry in the analyzer was measured with shoes off and using a wall-mounted stadiometer.

The Automated Self-Administered 24-h Dietary Assessment was completed by participants before enrollment to characterize baseline habitual intake. Each participant entered two 24-h recalls, 1 weekday and 1 weekend day, to capture variation in intake. The first recall was completed during the screening visit under staff guidance, and the second was completed at home before the start of the study.

#### FibroScan measurements

FibroScan transient elastography is widely used, noninvasive tool validated for estimating liver fat content through the controlled attenuation parameter (CAP) [29]. Liver stiffness was assessed by transient elastography (FibroScan, Echosens). The examination was performed at the Human Research Core at the University of North Carolina Chapel Hill, Nutrition Research Institute, Kannapolis, NC, by technicians or physicians blinded to the participant’s choline intake status. The FibroScan used was a FibroScan 502 Touch model, equipped with S, M, and XL probes. Probes were automatically selected by the software’s tool. The examination procedure was as follows: participants were asked to fast 1 night before the examination, then placed in a supine position with their right arm fully abducted. Participants were asked to hold their breath for 10–15 s, and ≥12 measurements per participant were collected on day 1 and day 15 of each arm. Measurements were performed by scanning the liver through an intercostal space. We repeated the procedure and recorded ≥4 sets of 12 measurements per collection day. The raw ultrasonic radio frequency signals were stored in the FibroScan file. Due to an unexpected software update, 7 FibroScan datasets were lost, 2 from the 25% AI arm, 4 from the 50% AI arm, and 1 from the 100% AI arm. CAP was computed only when the measurement was valid. The final CAP results were expressed in dB/m and corresponded to the average of valid individual measurements. Importantly, in our study, FibroScan was not used to diagnose steatosis [30,31], but rather to detect significant changes in liver fat content resulting from the choline dietary intervention.

### Mass spectrometry for plasma choline metabolites and urine riboflavin

Samples were prepared by spiking with stable isotope-labeled internal standards, followed by extraction using a protocol we established [23,27,32]. Briefly, each sample was combined with 4 volumes of methanol:chloroform (2:1, v/v), vortexed, and incubated at −20°C for 2–24 h before a second vortexing step. The resulting supernatant was collected, and the remaining pellet was subjected to a secondary extraction using methanol:chloroform:water (2:1:0.8, v/v/v). Supernatants from both steps were pooled and subjected to phase separation by the addition of water and chloroform. The aqueous layer, containing choline and betaine, was transferred to HPLC vials for analysis. The organic phase, containing PtdCho and sphingomyelin, was diluted in acetonitrile and similarly transferred to HPLC vials. Calibration standards containing known analyte concentration and internal standards were prepared in parallel and processed using the same extraction steps.

Quantification of analytes was performed by LC-SID-MRM/MS for both aqueous and organic fractions, as previously established [23,27,32]. Chromatographic separation was achieved using an Acquity HILIC column (1.7 μm, 2.1 × 50 mm; Waters Corp.) on a Waters ACQUITY UPLC system, operated at a flow rate of 0.37 mL/min and maintained at 40°C. For aqueous-phase analysis, the mobile phases consisted of *1*) 100% water containing 0.125% formic acid, and *2*) 90:10 acetonitrile:water with 10 mM ammonium formate and 0.125% formic acid. Mobile phase A for organic analytes was 10% acetonitrile/90% water and 0.125% formic acid and B was the same as for aqueous metabolites. As described previously [32], PtdCho undergoes in-source fragmentation and is detected in the form of phosphocholine. Metabolites and their corresponding isotope-labeled standards were monitored on a Waters triple quadrupole mass spectrometer, using specific precursor-product ion transitions. Analyte concentrations were calculated from the ratio of the signal intensity of each analyte to its internal standard, referenced against a standard curve generated from known concentrations.

Assay performance characteristics followed the validated LC-SID-MS method previously established by our laboratory [23]. Limits of detection (LOD) were defined as the concentration at which the signal was 3 times the SD of blank samples, and the lower limit of quantification (LLOQ) as 5 times the SD of blank samples. Our previously reported LODS for choline and betaine were 0.008 and 0.122 μM, with LLOQ of 0.122 and 0.488 μM, respectively. These thresholds were applied to analytes in this study. Values below the LOD were classified as not detected, and signals between the LOD and LLOQ were designated as trace and not used for quantitative analysis. All samples were analyzed in duplicate, and samples with >15% difference between duplicates were reextracted and reanalyzed. External quality control (QC) materials including pooled human plasma controls and matrix controls were processed in parallel. These QC materials demonstrated low coefficients of variation and acceptable deviation from expected concentrations, confirming assay precision and extraction consistency. Interassay and intra-assay coefficients of variation for this validated method are <10%, as previously reported.

The isotopic enrichment ratio (IER) was determined by calculating the proportion of labeled metabolite relative to the total (labeled plus unlabeled) pool, providing a measure of precursor availability within the metabolic pathway [27,33].

### Mass spectrometry for homocysteine

Total plasma homocysteine (tHcy), representing the sum of all reduced and oxidized forms after disulfide bond cleavage, was quantified using LC-ESI-MS/MS, as previously described with some minor modification [34]. Plasma (10 μL) was prepared by incubation on ice for 20 min with an aqueous solution (120 μL) containing internal standard d8-homocystine (Cambridge isotopes) and dithiothreitol. The mixture was then deproteinized by the addition of acetonitrile (400 μL), which was mixed and then centrifuged at 14, 000 × *g* for 10 min at 4°C. The resulting clear extract was transferred to a 96-well plate and loaded into a refrigerated autosampler for analysis by the mass spectrometry system (4000 QTRAP, AB Sciex LLC). Data were acquired and processed using Sciex Analyst v1.6.24 (AB Sciex LLC).

### Receiver operating characteristic data analysis

Receiver operating characteristics (ROC) curves were generated in R using the boot and pROC packages [35–37], and only complete paired observations from participants who completed both the 25% AI and 100% AI arms were included. For the combined choline + betaine analysis, a logistic regression model was fitted, and the resulting predicted probabilities were used as the continuous predictor for ROC analysis. To account for model-development uncertainty arising from fitting and evaluating the model on the same dataset, a stratified nonparametric bootstrap resampling (2000 replicates), stratified by outcome group (25% AI compared with 100% AI), was performed. Within each bootstrap resample, the combined model was refitted, and the difference in area under the curve (AUC) between the combined model and individual metabolites was calculated. In addition, a Wald-type test was performed using a bootstrap-estimated SE to compute a Z statistic and 2-sided *P* value.

ROC analysis was applied to test the ability of plasma choline and betaine to discriminate between low (25% AI) and adequate (100% AI) choline intake levels. This approach was selected because ROC analysis provides a nonparametric, threshold-independent measure of discriminatory performance and is widely used to evaluate response indicators in nutrition studies [38]. Because the outcome represented assigned dietary conditions rather than predicted probabilities, the analysis focused on discrimination rather than calibration [39].

### Statistical analysis

We conducted a repeated measures mixed-effects analysis with Geisser-Greenhouse correction using GraphPad Prism 10 (GraphPad Software Inc). This model is well-suited for our randomized design and accommodates random missing values without the need for imputation, making it preferable to the traditional ANOVA context. Prism implements this model using restricted maximum likelihood (REML) estimation and treats each participant as a repeated-measure factor. Normality of residuals and model assumptions were evaluated by inspecting residual compared with fitted plots and quantile–quantile plots generated in Prism. For multiple comparisons, we used Tukey’s test. False discovery rate adjustment was applied only in the isotope enrichment analyses, where families of related post hoc contrasts required correction, and adjustments were performed within each modeled endpoint rather than across days and IER calculations.

The mixed-effects framework was applied to the targeted plasma metabolite outcomes, isotope dilution enrichment ratios, AST, ALT, and FibroScan outcomes, and naturally accommodates occasional missing observations without requiring imputation.

Linear mixed-effects modeling was performed in R (version 4.3.0) using lme4 (version 1.1–35.3) and lmerTest (version 3.1–3) packages [40]. Plasma concentrations of choline and choline-related metabolites were modeled as the continuous outcome variables. Fixed effects included choline intake (modeled as 3 levels: 100% AI, 50% AI, and 25% AI) and sex/hormonal status, represented as a 4-factor categorical variable (male, female-premenopausal, female-perimenopausal, and female-postmenopausal). To account for the correlation between repeated measures or multiple observations from the same individual, a random intercept was included for participant ID, capturing individual-level variation in baseline metabolite concentrations. *P* values for fixed effects were obtained using Satterthwaite’s approximation for degrees of freedom, as implemented in the lmerTest package. All models were fit using REML estimation [41]. *P* values < 0.05 were considered significant.

ROC analyses were performed in R using the pROC package, which does not permit missing values and therefore includes only participants with complete paired measurements for both the 25% AI and 100% AI choline intake conditions. A total of 71 participants (94%) had complete paired measurements at both 25% AI and 100% AI plasma choline and were included in the ROC analysis; the remaining 4 participants lacked 1 of the 2 measurements and were therefore excluded.

Primary analysis (plasma metabolite concentrations, isotopic enrichment rations, and FibroScan), as well as subgroup analyses by sex and menopausal status, were prespecified and conducted on blinded data. Post hoc exploratory analysis included FibroScan responders, regression modeling, and ROC curve modeling.

## Results

Of the 140 individuals assessed for eligibility, 44 were excluded (31 declined participation, 8 did not meet inclusion criteria or had medical issues, and 5 participated only in an early pilot phase). A total of 96 participants were enrolled and randomly assigned. Withdrawals occurred across the dietary arms for personal reasons (*n* = 21) and noncompliance (*n* = 3), and 2 additional participants completed only the first 2 arms (Figure 1). Baseline characteristics of the randomized cohort are shown in Table 1.

Plasma choline concentrations are tightly regulated and may not reliably reflect dietary choline intake on their own [17]. To address this limitation, we evaluated whether the combined measurement of plasma choline, betaine, and PtdCho concentrations could more accurately reflect dietary choline intake.

### Isotopic enrichment ratios of betaine respond as a function of the dietary choline intake after a d_9_-choline bolus

Plasma concentration of choline, betaine, and PtdCho concentrations were measured by MS at baseline (prebolus; d_0_) and 24-h postbolus (d_9_) after administration of d_9_-choline bolus. IER were calculated as the concentration ratio of d_9_-metabolite to (d_0_ + d_9_-metabolite), reflecting the proportion of labeled compound in the circulating pool. Metabolite concentrations are presented in Table 2. Due to the absence of detectable d_9_-choline in plasma at 24 h, these data are included in Supplementary Table 3.

A mixed-effect analysis adjusted for false discovery rate revealed a significant diet-dependent effect on baseline d_0_-choline and betaine concentrations (*q* < 1 × 10^−4^) but not for PtdCho. Pairwise comparisons for PtdCho were nonsignificant across dietary groups: 25% AI compared with 50% AI (*q* = 0.277), 25% AI compared with 100% AI (*q* = 0.531), and 50% AI compared with 100% AI (*q* = 0.533) (Table 2 and Supplementary Table 3). These findings indicate that baseline plasma concentrations of choline and betaine, but not PtdCho, reflect dietary choline intake.

For the labeled metabolites, d_9_-betaine showed a strong intake-dependent response (overall *q* < 1 × 10^−4^), with significant pairwise differences between 25% AI and both 50% AI (*q* = 0.004) and 100% AI (*q* < 1 × 10^−4^), as well as between 50% AI and 100% AI (*q* = 0.003).

d_9_-PtdCho also varied by intake level (*P* = 0.001), with significant differences observed between 25% AI compared with 100% AI (*q*=0.001), and 50% AI compared with 100% AI (*q* =6 × 10^−4^), but not between 25% AI compared with 50% AI (*q* =0.265).

The values for IER-betaine demonstrated a significant overall effect of diet (*P* = 0.026), with differences between 25% AI compared with 50% and 100% AI (both *q* < 0.01), but not between 25% AI compared with 50% AI.

IER-PtdCho showed a significant overall effect on the mixed-effects model (*P* = 1 × 10^−4^). Pairwise comparisons indicated highly significant differences between 25% AI compared with 100% AI and 50% AI compared with 100% AI, we found highly significant differences (*q* = 1 × 10^−4^and *q* = 5 × 10^−4^, respectively) and not significant differences between 25% AI compared with 50% AI (*q* = 0.126).

Together, these findings confirm that d_9_-betaine and d_9_-PtdCho, and their respective IERs, are responsive to dietary choline intake, particularly in comparisons between the 25% AI and 100% AI groups. These results are aligned with previous pilot findings [27], now supported by an increased number of participants. Because the above analyses reflect the response to a single d_9_-choline bolus administered on day 12 and evaluated at day 13, we next assessed whether steady-state plasma concentrations of choline, betaine, PtdCho and tHcy at the end of each arm (day 15) also reflected dietary intake by using targeted metabolomics.

### Plasma choline and betaine concentrations reflect dietary choline intake

Using targeted MS, we assessed plasma concentrations of choline, betaine, PtdCho, and tHcy on day 15, marking the end of each study arm. Because choline metabolism differs between males, premenopausal females and menopausal females based on sex and hormonal status [17], our evaluations consider these factors.

**FIGURE 2.**
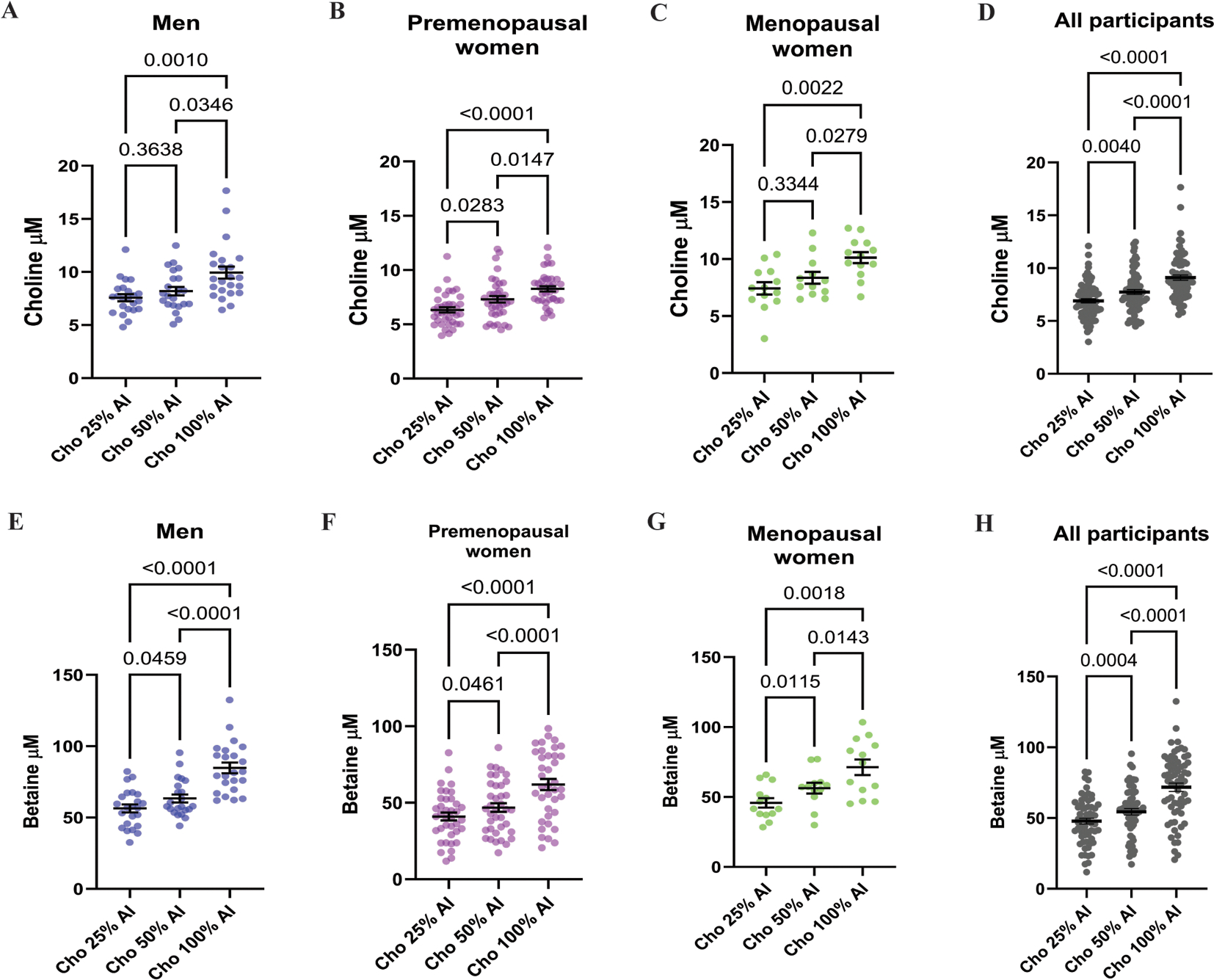
Plasma choline and betaine concentrations by sex and dietary choline intake level. Plasma choline concentrations (A–D) and plasma betaine concentration (E–H) are shown at the end of each dietary arm (25%, 50%, and 100% of the AI for choline). Graphs show data stratified by sex and menopausal status, males, premenopausal females, menopausal females and all participants. Each dot represents 1 participant; bar represents the group mean ± SEM. Data were analyzed using a linear mixed-effects model with fixed effect for diet and subject as a random effect with Geisser-Greenhouse correction. Tukey’s post hoc test with adjusted *P* values was used for multiple comparisons. Sample size: males *n* = 23, premenopausal females *n* = 38 and menopausal females *n* = 13. Note that for the arm Cho 50% AI, plasma samples could not be obtained for 1 male and 1 menopausal female. Perimenopausal female included in “all participants.” AI, adequate intake; Cho, choline.

We observed that plasma choline concentrations were statistically significant across dietary choline intake levels (25%, 50%, and 100% AI) in males, with the exception of the comparison between 25% and 50% AI (Figure 2A). In premenopausal females, significant differences were also detected across the choline intake levels (Figure 2B). However, in menopausal females, a significant difference was only observed between the 25% AI and 100% AI groups (Figure 2C). When data from all participants were combined, plasma choline concentrations differed significantly and in the same direction as the dietary choline intake (Figure 2D).

As described in the methods, all study diets were designed to provide approximately equal amounts of betaine (~147 mg betaine/d). Therefore, observed changes in plasma betaine concentrations are a response to differences in dietary choline intake. By day 15, plasma betaine levels varied significantly across the intake levels in all the groups, except for males comparing 25% AI to 50% AI (Figure 2E). In both premenopausal and menopausal females, betaine concentrations differed significantly by dietary intake level (Figure 2F–G). When data were analyzed across all participants, significant differences in plasma betaine were also observed in response to dietary choline intake (Figure 2H).

In contrast, plasma PtdCho concentrations at day 15 did not differ significantly across choline intake levels when stratified by sex, hormonal status, or in the overall cohort (Supplementary Figure 1A–D). Given that both choline and betaine are inversely associated with plasma tHcy concentrations [42,43], we measured tHcy at day 15 of each dietary arm (Supplementary Figure 1E–H). A statistically significant reduction in tHcy was observed when comparing the 25% AI compared with 50% AI groups (*P* = 0.033) in the combined all participants analysis; however, no significant differences were detected in other comparisons. Collectively, these findings support that plasma choline and betaine concentrations are responsive to dietary choline intake across the full cohort and within subgroups defined by sex and hormonal status.

### Fibroscan detects variable liver fat responses to dietary choline intake

We then asked whether additional clinical markers could enhance the prediction of dietary choline intake. We previously demonstrated that severe dietary choline restriction, specifically 10% of the AI, was associated with the development of steatosis and liver injury [16,17]. In the current study, the lowest choline intake was 25% of AI. Given this, we investigated whether this level of choline restriction was sufficient to elicit early markers of liver dysfunction. To assess liver function, we measured the circulating AST and ALT concentrations at the end of each arm. No statistically significant differences were observed among the 3 dietary choline groups (Supplementary Figure 2A–H). These findings suggest that choline intake >25% of the AI, when consumed >15 d, were sufficient to prevent elevation in liver enzymes. This contrasts with our prior study in which prolonged consumption, ≤7 wk, of diets providing only 10% of the AI led to clinically evident liver injury [16,17].

Because we did not observe abnormal liver enzymes in circulating plasma, we tested whether FibroScan, a noninvasive approach to assess liver fat [44], could detect liver fat changes in response to the different choline dietary intakes. We measured liver fat as CAP. We calculated the final value for each arm by averaging 4 independent sets, each consisting of 12 measurements. First, we analyzed whether participants exhibited significant differences on day 1 of each arm, with the objective of correcting liver fat differences at the start of the study. We analyzed results stratified by sex and hormonal status, and we did not find differences on day 1 when comparing CAP values between 25% AI compared with 50% AI compared with 100% AI (Supplementary Figure 3A–C). However, when we pooled all the participants, we found significant differences on day 1 when starting the 50% AI compared with 25% AI arm (*P* = 0.012; Supplementary Figure 3D), suggesting that baseline liver fat was influenced by their regular dietary intake before the study enrollment. As a result, we focused our analysis on day 15 CAP measurements, after participants had consumed controlled diets for 15 consecutive days.

When analyzing the impact of consuming 50% AI choline diet, we observed a highly variable response; some participants showed hepatic fat accumulations whereas others remained unaffected (Supplementary Figure 3E). These interindividual differences in response to choline restriction are likely to be multifactorial and may, in part, be driven by genetic variation influencing choline metabolism [45–47].

**FIGURE 3.**
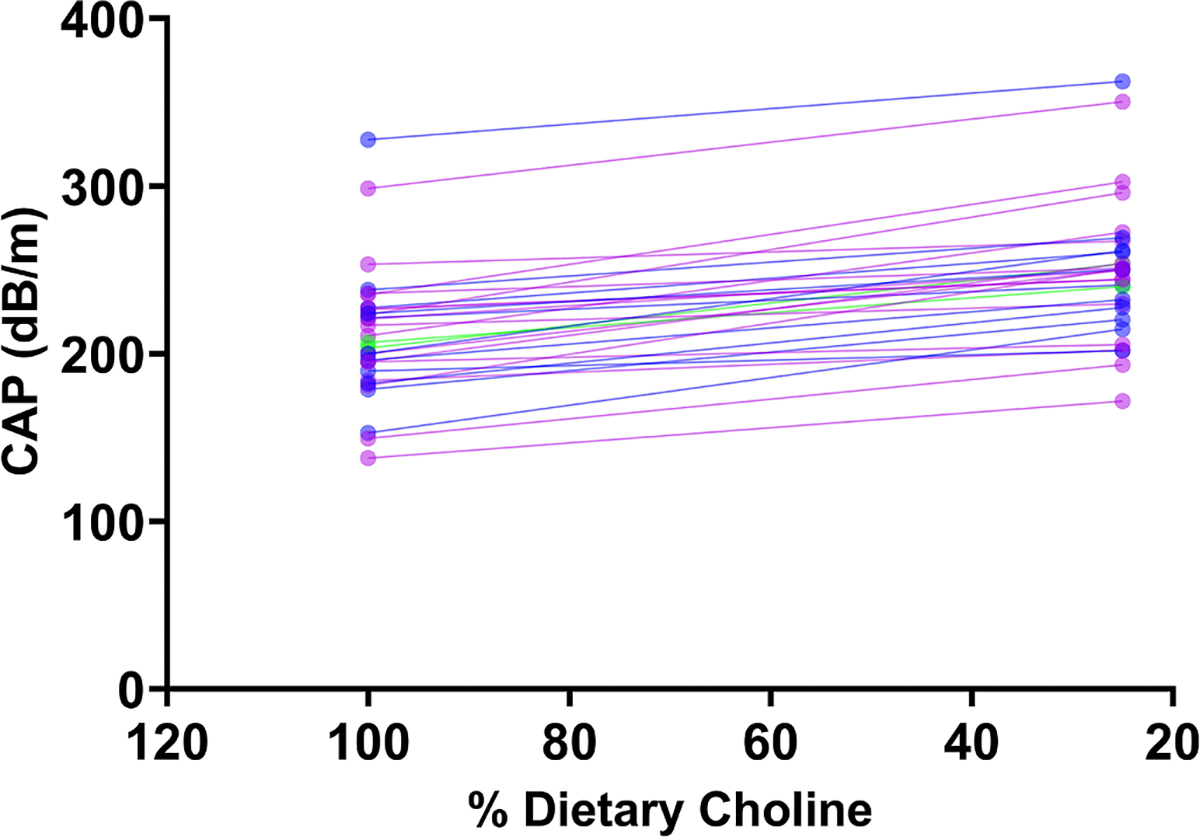
Individual slope plots showing changes in CAP measured by FibroScan at the end of the 100% AI and 25% AI dietary choline intake. Each line represents 1 participant, with CAP values (dB/m) measured at the end of each condition. These participants showed ≥10% increase in liver fat when consuming 25% AI compared with 100% AI. AI, adequate intake; CAP, controlled attenuation parameter.

Given our previous results, we asked if there was an increase in liver fat accumulation from 100% AI compared with 25% AI arms. Participants were classified as responders if they exhibited a ≥10% increase in CAP values between the 100% AI arm compared with 25% AI. For this analysis, we used participants with complete FibroScan assessments. Among 72 participants, 30 individuals (41.6%), exhibited an increase of ≥10% in liver fat when consuming a 25% AI diet (Figure 3); this group was classified as “responders,” and the rest of the participants did not meet this criterion (Supplementary Figure 3F). To assess whether these classifications were consistent across dietary arms, we compared participant responses between the 50% and 25% AI diets. The groups were not identical, only 12 participants showed a reproducible ≥10% increase in liver fat across both the 50%→25% and 100%→25% AI comparisons. The 50% AI arm was particularly variable for some participants; this level of choline intake may have been sufficient to challenge hepatic lipid handling, whereas others appeared able to compensate.

Altogether, these findings indicate that liver fat serves as a marker of dietary choline intake in a specific subset of participants who demonstrate an increase in CAP averages when consuming 25% AI choline. However, the utility of FibroScan as a standalone tool for assessing dietary choline intake is limited, given that a considerable portion of participants exhibited no or minimal changes in liver fat across choline dietary intake.

### Association between dietary choline intake and liver fat content in a linear mixed-effects model

Because we had plasma choline metabolites and FibroScan CAP numbers at the end of each arm, we conducted linear regression analysis to assess the effect of dietary choline intake (25% AI and 50% AI) compared with the 100% AI reference group on plasma metabolites (choline, betaine, PtdCho and tHcy) and CAP values as a reflection of liver fat. Both plasma and betaine concentrations were significantly lower in 25% AI and 50% AI groups relative to 100% AI. In contrast, PtdCho concentrations did not differ significantly by dietary choline intake level (Table 3).

tHcy was modestly elevated in the 25% AI group which is consistent with previous reports where total choline and betaine intake was inversely associated with tHcy [42]. No statistically significant differences in liver fat, as measured by Fibroscan CAP values at day 15, were observed across the groups. Similarly, a change in CAP (ΔCAP) did not differ significantly between groups (Supplementary Table 4). To verify that the randomized diet sequence did not influence outcomes, we also tested for potential carry-over effects by including arm order as a fixed factor in the linear mixed-effects models. No significant effects of diet sequence were detected for any metabolite or liver outcome (Supplementary Table 5). These results indicate that plasma choline and betaine concentrations are sensitive to moderate changes in dietary choline intake.

### Use of plasma choline and betaine concentration shows high discrimination in classifying dietary choline intake

Across multiple analytical approaches shown in this study, our findings consistently demonstrated that plasma choline and betaine concentrations responded to dietary choline intake, with the most pronounced differences observed between the lowest (25% AI) and highest (100% AI) choline intake levels. To further evaluate the utility of these metabolites in discriminating between dietary choline intake, we performed receiver operator characteristics (ROC) curve analysis using plasma values from participants completing the 25% AI to 100% AI arms.

**FIGURE 4.**
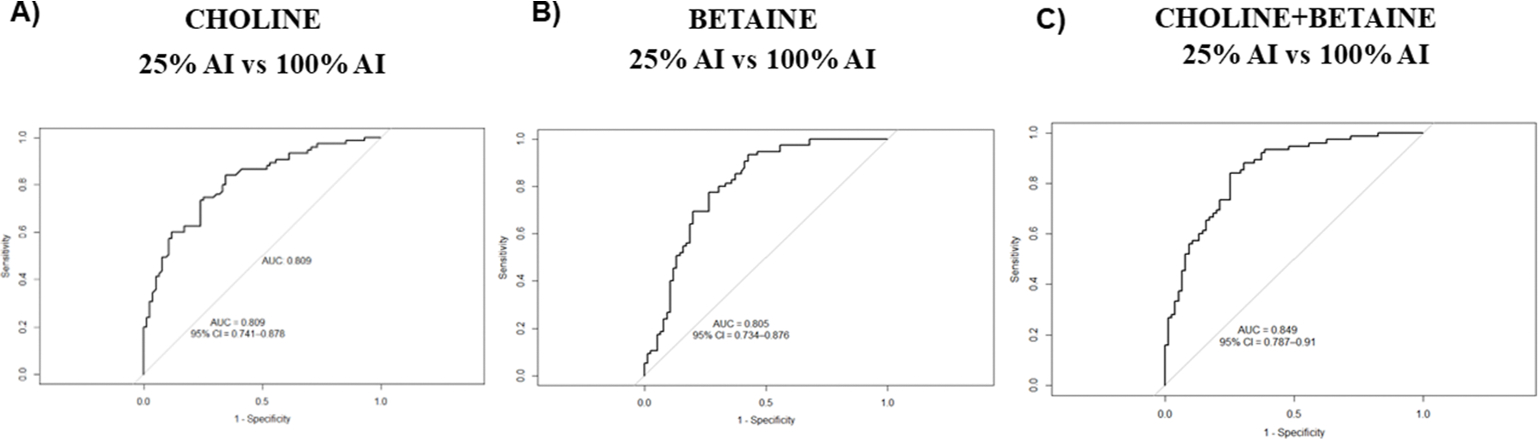
ROC curve showing the ability of plasma choline, betaine, and their combination to discriminate 25% compared with 100% AI choline intake. (A) ROC curve for plasma choline as a discriminator of dietary choline intake. The AUC was 0.81 (95% CI: 0.74, 0.88), indicating good discriminatory capacity. (B) ROC curve for plasma betaine as a discriminator of dietary choline intake. The AUC was 0.80 (95% CI: 0.73, 0.88), indicating good discriminatory ability. (C) ROC curve evaluating the performance of choline and betaine in reflecting dietary choline intake. The AUC was 0.85 (95% CI: 0.79, 0.91), indicating excellent discrimination (*n* = 71 participants). Statistical analyses were performed in R using the pROC package. AI, adequate intake; CI, confidence interval; ROC, receiver operating characteristic.

Plasma choline alone yielded an AUC of 0.81 [95% confidence interval (CI): 0.74, 0.88] indicating a good discriminatory capacity (Figure 4A). Similarly, plasma betaine exhibited an AUC of 0.80 (95% CI: 0.73, 0.88) (Figure 4B), supporting its role as an individual predictor. When choline and betaine were combined, the AUC increased to 0.85 (95% CI: 0.79, 0.91), indicating excellent discrimination with a CI spanning from acceptable to outstanding discrimination (Figure 4C). Formal comparison using bootstrap-based Wald tests showed that the combined choline + betaine model demonstrated a modest, borderline improvement in discrimination relative to betaine alone (ΔAUC = 0.043, *P* = 0.055), with a similarly small and nonsignificant improvement relative to choline alone (ΔAUC = 0.039, *P* = 0.080).

These findings suggest that choline and betaine, alone and in combination, are robust discriminators of dietary choline intake, specifically distinguishing low choline intake (25% AI) from AI (100% AI). We acknowledge that a limitation of our approach is the lack of independent manipulation of dietary betaine, which may contribute to collinearity in the predictive model. Nonetheless, the consistency and strength of the ROC performance support the value of these markers in assessing choline dietary intake under controlled conditions.

## Discussion

The role of choline in human nutrition is well-established, particularly in relation to brain and cognitive function, liver metabolism, and muscle health [2,7,18,45,48]. Despite this, accurate determination of dietary choline intake remains a methodological challenge. Early studies demonstrated that consuming only 10% of the AI of choline for 6 wk can result in the development of hepatic steatosis and liver injury [17]. However, the effects of a moderate reduction in choline intake over a shorter duration may provide a more realistic insight into habitual dietary choline intake patterns. With this perspective in mind, the present study employed a combination of approaches to identify early and sensitive indicators of low dietary choline intake that could support more refined dietary guidance. To contextualize these findings, it is also important to consider the strengths and limitations of plasma choline and betaine as discriminators of low dietary choline intake and how they compare to other metabolites that have been proposed in prior work.

In our first approach, we administered a single bolus of deuterium-labeled choline (d_9_-choline chloride) as a tracer to evaluate whether plasma levels of d_9_-choline, d_9_-betaine or d_9_-PtdCho, measured 24 h post dose, reflected dietary choline intake. Although d_9_-betaine or d_9_-PtdCho were reliably detected and demonstrated significant differences, d_9_-choline was not detectable in most participants at the 24-h post bolus (the liver rapidly clears choline from blood after oral administration [49]). These findings are consistent with our earlier interim analysis during method development where 6 h post bolus showed modest changes, and the values were low and showed minimal discrimination by intake level [27]. Although choline chloride differs from food-derived PtdCho in solubility and absorption route, both ultimately converge into shared hepatic pools. After absorption, choline chloride is rapidly phosphorylated and incorporated into PC and PtdCho in the liver, from which it is gradually released into circulation in a manner that parallels dietary PtdCho.

d_9_-PtdCho concentrations in plasma were comparatively high, though they showed weaker discrimination across intake levels. This metabolic partitioning is consistent with known kinetics of choline utilization, at lower concentrations of choline, the liver phosphorylates it and produces PtdCho; at higher concentrations of choline, choline kinase becomes saturated and most choline is oxidized to form betaine [49]. Notably, as outlined in the Methods section, we calculate the same amount of betaine intake in the diets provided, enhancing our confidence in attributing changes to dietary choline intake.

Previously, tracer experiments in males who consumed d_9_-choline revealed that this method can discern choline metabolism variations driven by genetic variants in the methylenetetrahydrofolate reductase (MTHFR) (677CC or 677TT) gene [50]. One of the major contributions of our approach is the potential for reflecting dietary choline intake with a single bolus of d_9_-choline chloride, irrespective of sex or genotype. Consolidating these findings highlights the necessity of evaluating established genetic variants linked to increasing the requirements [51,52]. However, the cost of performing tracer experiments limits its use as a reflector of recent dietary choline intake in regular practice.

To further investigate the role of choline metabolites in plasma samples as indicators of diet intake, we conducted targeted quantification of choline, betaine, PtdCho, and tHcy, on day 15 of each arm. Our findings also identified choline and betaine concentrations as the primary markers closely reflecting dietary choline intake. Previous research showed that choline supplementation (930 compared with 480 mg/d) increases the plasma concentrations of PtdCho in nonpregnant females [53]. In our study, plasma PtdCho concentrations did not exhibit a significant association with dietary choline intake and we speculate that 25% AI choline dietary intake for 15 d is not sufficient to reduce the circulating concentration [54–56]. Alteration in plasma tHcy concentration had been inversely associated with both choline and betaine dietary intake when folate and vitamin B12 concentrations were low [57], but we did not see this association in our study, where these latter nutrients did not vary between the diet arms.

The use of FibroScan to assess liver fat content revealed a substantial interindividual variability, limiting its utility as a standalone predictor of choline intake. Although a subset of participants exhibited increased liver fat following the 25% AI diet, this response was not consistently observed among those with the lowest circulating choline concentrations. These findings align with our previous use of MRS to quantify hepatic total soluble choline species, where the association with dietary choline was similarly nonsignificant (*q* = 0.08) and driven by interindividual variability [27]. Together, these results suggest that the hepatic response to short-term choline restriction is highly individualized and that imaging methods alone may be insufficient to capture subtle metabolic effects. It is also important to consider that FibroScan was originally developed for clinical evaluation of liver fibrosis and steatosis, and it is optimized for detecting moderate-to-severe steatosis (S0–S3) [58,59]. Moreover, the 15-d duration of low choline intake may not have been sufficient to induce detectable changes by FibroScan. The timescale for detectable hepatic responses to choline restriction extends beyond the 15-d period examined here. In choline depletion studies, increases in liver fat and aminotransferases appeared only after 3–6 wk of near-zero choline intake, whereas even 12 wk of moderate restriction (300 mg/d) in males carrying the *MTHFR C677TT* or *CC* genotype caused no enzyme change [60], indicating that both longer duration and more severe depletion are required before group-level FibroScan or biochemical alterations become evident, although individual susceptibility may lead to earlier hepatic responses in some participants.

In this context, the absence of changes in serum aminotransferases further indicates that classical liver enzymes lack sensitivity for detecting short-term effects of a low choline diet. Future studies should therefore integrate FibroScan with exploratory metabolic markers, such as circulating indices of de novo lipogenesis or lipidomic profiles, which may offer greater sensitivity for detecting low choline–induced hepatic lipid changes.

Regression modeling provided additional support for plasma choline and betaine as metabolites that respond to changes in dietary choline intake. In contrast, PtdCho and liver CAP values did not show significant variation. As discussed earlier, this may reflect the relatively short duration of the choline diets or the influence of metabolic compensation mechanisms. Our findings highlight the value of assessing multiple outcomes in parallel to strengthen data interpretation.

The use of a multivariate ROC analysis as a binary classification tool showed that choline and betaine together provided excellent discrimination of dietary choline intake. This analysis was intended to evaluate classification performance rather than to model absolute risk and demonstrated that the combined metabolites offered a slightly stronger discrimination than either metabolite alone. Notably, the CI of the combined model extended into the outstanding range, reinforcing the strength of this combined approach. A limitation of this approach is that plasma choline and betaine may be influenced by hormonal status and genetic variation in 1-carbon metabolism which could reduce discriminative performance outside controlled feeding conditions.

Beyond the targeted metabolites examined here, prior metabolomics studies of severe choline depletion have shown coordinate and consistent changes in acylcarnitines [16]. These pathways reflect broader metabolic responses to choline insufficiency but consistent sensitivity to moderate variations in intake needs to be tested.

The present work provides a cost-effective and accessible method to discriminate low dietary choline intake, requiring only plasma measurements of choline and betaine and substantially less time consuming compared with traditional tracer studies. Next steps should aim to validate this approach in free-living environments and expand the study to encompass a larger population, including for instance a larger representation of menopausal females, given the well-established influence of hormonal status on dietary choline intake [14,15].

In summary, our findings demonstrate that plasma choline and betaine are responsive to controlled variations in dietary choline intake, supporting their utility as predictors of dietary intake via ROCs. The discriminatory capacity of these metabolites was strongest when distinguishing low intake (25% AI) from AI (100% AI), highlighting their value as indicators of insufficient choline consumption rather than as part of a continuous dose–response relationship. Importantly, these results underscore the need to expand future research to include genetic variants in choline metabolism and hormonal status as key factors in evaluating individual choline requirements.

## Supplementary Material

1

## Figures and Tables

**TABLE 1:** Participant characteristics.

	Males	Premenopausal females	Menopausal females	Perimenopausal females

*n*	24	39	14	1
Age (y)	36 ± 9.7	35 ± 8.5	53 ± 6.8	49
Height (cm)	177 ± 6	165 ± 5	166 ± 5.8	161
Weight (kg)	77 ± 9	70 ± 6.9	68 ± 7.8	74.20
BMI (kg/m^2^)	24 ± 2	26 ± 2.8	25 ± 3	28.60
Ethnicity (White/Black/Hispanic)	21/2/1	26/7/6	11/3/0	1/0/0
FSH (mIU/mL)	—	6 ± 3.30	79. ± 31.4	23
Estrogen (mIU/mL)	—	212 ± 150.6	96.79 ± 66.5	128

Values are mean ± SD, ranges, or proportions. FSH and estrogen concentrations were measured by LabCorp and were used to classify hormonal status. Table 1 reflects the 78 participants who completed enrollment procedures. Three participants were excluded from analysis due to noncompliance (1 male, 1 female, and 1 menopausal female). Analytical sample sizes for individual outcomes were smaller due to lost samples, as detailed in Methods and Figure legends.

FSH levels as measured by LabCorp were used to classify menopausal status. Abbreviation: FSH, follicle-stimulating hormone.

**TABLE 2:** Concentration of plasma betaine and PtdCho measured by mass spectrometry.

	Choline diet 25%	Choline diet 50%	Choline diet 100%

d_0_-betaine (μm)			
Males	63.5 ± 10.3	74 ± 13	92.5 ± 18
Premenopausal (F)	45 ± 15	52 ± 18	68 ± 25
Menopausal (F)	53.4 ± 12	61.8 ± 15	80.5 ± 23
All	52 ± 15	61 ± 18	78 ± 25
d_9_-betaine (μm)			
Males	1.6 ± 0.47	1.9 ± 1.20	2.0 ± .77
Premenopausal (W)	1.18 ± 0.68	1.33 ± 0.67	1.63 ± 0.80
Menopausal (W)	1.26 ± 0.66	1.44 ± 0.71	1.73 ± 0.80
All	1.3 ± 0.64	1.6 ± 0.92	1.8 ± 0.81
IER-betaine (μm)			
Males	0.024 ± 0.004	0.025 ± 0.010	0.021 ± 0.006
Premenopausal (W)	0.023 ± 0.680	0.024 ± 0.006	0.022 ± 0.005
Menopausal (W)	0.022 ± 0.008	0.021 ± 0.010	0.020 ± 0.005
All	0.022 ± 0.008	0.024 ± 0.008	0.002 ± 0.005
d_0_-PtdCho (μm)			
Males	2055 ± 408	2132 ± 481	2056 ± 424
Premenopausal (W)	2172 ± 474	2240 ± 526	2208 ± 551
Menopausal (W)	2583 ± 421	2636 ± 453	2707 ± 607
All	2209 ± 476	2277 ± 524	2249 ± 565
d_9_-PtdCho (μm)			
Males	27.1 ± 7.02	26.8 ± 9.74	21.8 ± 7.94
Premenopausal (W)	24.3 ± 8.36	25 ± 7.92	22.6 ± 5.88
Menopausal (W)	25.9 ± 6.82	26.4 ± 10.6	25 ± 3.85
All	26 ± 7.7	26 ± 8.9	23 ± 6.4
IER-PtdCho (μm)			
Males	0.013 ± 0.002	0.012 ± 0.003	0.010 ± 0.002
Premenopausal (W)	0.011 ± 0.003	0.011 ± 0.002	0.010 ± 0.002
Menopausal (W)	0.010 ± 0.003	0.010 ± 0.003	0.009 ± 0.001
All	0.012 ± 0.003	0.011 ± 0.003	0.010 ± 0.002

Betaine and PtdCho were measured by MS at basal and 24 h post bolus (2.2 mmol d_9_-choline). Values are mean ± SEM. Statistical comparisons were performed using a linear mixed-effects model with FDR correction (Benja-mini–Yekutieli). Data for the single perimenopausal female are included in all but not listed separately. Males *n* = 23, menopausal females *n* = 13, premenopausal females *n* = 38, and perimenopausal female *n* = 1.

Abbreviations: FDR, false discovery rate; IER, isotopic enrichment ratios; MS, mass spectrometry; PtdCho, phosphatidylcholine.

**TABLE 3:** Linear mixed-effects model estimates (*β* coefficients and 95% confidence intervals) comparing circulating metabolite concentrations of choline, betaine, and PtdCho across dietary choline intake levels.

Metabolite	β (LCL, UCL)	*P* value

Choline		
Ref: 100% AI	—	—
25% AI	−2.20 (−2.72, −1.68)	5.44 × 10^−14^
50% AI	−1.35 (−1.87, −0.83)	1.20 × 10^−6^
Betaine		
Ref: 100% AI	—	—
25% AI	−24.05 (−27.72, −20.39)	1.1 × 10^−25^
50% AI	−16.82 (−20.52, −13.12)	2.0 × 10^−15^
Ptd choline		
Ref: 100% AI	—	—
25% AI	−38.22 (−255.79, 179.09)	7.3 × 10^−1^
50% AI	37.23 (−181.83, 256.32)	7.39 × 10^−1^

The 100% AI condition was used as the reference group. Fixed effects included choline intake and sex/hormonal status, whereas participant ID was included as a random intercept to account for repeated measures. Plasma choline and betaine concentrations were significantly lower in the 25% and 50% AI groups compared with 100% AI.

Abbreviations: AI, adequate intake; CI, confidence interval; ID, identity document; LCL, lower control limit; PtdCho, phosphatidylcholine; UCL, upper control limit.

## Data Availability

Data described in the manuscript will be made available through the University of North Carolina Dataverse repository URL: https://doi.org/10.15139/S3/J17LGU.

## Appendix A. Supplementary data

Supplementary data to this article can be found online at https://doi.org/10.1016/j.ajcnut.2026.101236.

## Abbreviations

AI: adequate intake
ALT: alanine aminotransferase
AST: aspartate aminotransferase
CAP: controlled attenuation parameter
CK: creatine kinase
FSH: follicle-stimulating hormone
IER: isotopic enrichment ratios
LC: liquid chromatography
LOD: limits of detection
LLOQ: lower limit of quantification
MRS: magnetic resonance spectroscopy
MS: mass spectrometry
MTHFR: methylenetetrahydrofolate reductase
QC: quality control
PEMT: phosphatidylethanolamine N-methyltransferase
PtdCho: phosphatidylcholine
REML: restricted maximum likelihood
ROC: receiver operator characteristics
SID-MRM: stable isotope dilution–multiple reaction monitoring
SNP: single-nucleotide polymorphism
SST: serum-separating tubes
tHcy: total homocysteine
