# Supplementary material for: Choline and betaine concentrations in plasma discriminate levels of dietary choline intake in healthy adults: analysis of a double-blind randomized crossover controlled feeding study
Source: Am J Clin Nutr. Author manuscript; Available in PMC 2026 May 5. (PMC13084590; doi:10.1016/j.ajcnut.2026.101236)

Choline and betaine concentrations in plasma discriminate levels of dietary choline intake in healthy adults: analysis of a double-blind randomized crossover controlled feeding study.

Isis Trujillo-Gonzalez et al.

Supplementary Table 1

Overview of the nutrient composition across the three base diets. Participants followed a daily rotation of three commercially prepared diets over each 15 day phase to minimize dietary monotony. Average nutrient values over three days, inclusive of choline supplementation, are reported. Optional snack choices—such as coffee, soda, gelatin, rice, or pears—were offered to help meet energy needs without meaningfully altering choline or betaine intake. All participants were required to consume lettuce and three bread rolls per day; the rolls included riboflavin as a compliance marker and contained any assigned supplemental choline (as choline chloride). Daily micronutrient supplementation consisted of one Kirkland Signature Daily Multi, one Nature Made Magnesium 250 mg, and two T.RQ Calcium 500 tablets per day. If a participant's chosen snacks did not fulfill their protein targets, a protein supplement was provided. Choline and betaine were quantified in our lab from homogenized and extracted foods using standard choline and betaine MS assay methods.

Day	Meal	Item(s)	Choline (mg)	Betaine (mg)	Energy (kcal)	Protein (g)	Carbohydrate (g)	Fat (g)
Day 1	Breakfast	Jimmy Dean Delights Croissant - Turkey sausage egg whites and cheese	33	22	290	16	30	13
	Lunch	Banquet Chicken Pot Pie	30	29	350	12	33	19
	Dinner	Amy's Enchilada Verde	61	7	400	17	54	13
	Optional items	Fruit, drinks, rice	7	1	675	8	166	1
	Mandatory items	Rolls (3), lettuce	23	50	331	10	57	7
	Daily total		154	109	2046	63	340	53
Day 2	Breakfast	Jimmy Dean Delights Croissant - Turkey sausage egg whites and cheese	33	22	290	16	30	13
	Lunch	Amy's Pesto Tortellini Bowl	36	51	450	17	50	20
	Dinner	Evol Butternut Squash Lasagna	23	20	430	20	56	17
	Optional items	Fruit, drinks, rice	7	1	675	8	166	1
	Mandatory items	Rolls (3), lettuce	23	50	331	10	57	7
	Daily total		122	144	2176	71	359	58
Day 3	Breakfast	Jimmy Dean Delights English Muffin - Applewood smoked chicken sausage egg whites and cheese	31	48	280	17	32	9
	Lunch	Lean Cuisine BBQ Chicken Pizza	42	36	390	21	55	9
	Dinner	Amy's Spinach Béchamel Lasagna	37	54	390	15	42	18
	Optional items	Fruit, drinks, rice	7	1	675	8	166	1
	Mandatory items	Rolls (3), lettuce	23	50	331	10	57	7
	Daily total		140	189	2066	71	352	44
		Low Choline (LC)	0	0	0	0	0	0

Choline supplement (as choline chloride) included in rolls	Moderate Choline (MC)	117	0	0	0	0	0
	High Choline (HC)	392	0	0	0	0	0
**3-day Average (LC)**		**139**	**147**	**2096**	**68**	**350**	**52**
**3-day Average (MC)**		**256**	**147**	**2096**	**68**	**350**	**52**
**3-day Average (HC)**		**531**	**147**	**2096**	**68**	**350**	**52**

Supplementary Table 2

Composition of rolls. Each batch of 120 rolls was prepared on-site, with ingredient quantities specified both per batch and per individual roll. *Flour was measured using a standard conversion of 120 grams per cup, based on King Arthur All purpose flour guidelines. Choline chloride was incorporated into the dough according to the procedure outlined in the Methods section.

	**Per 120 rolls:**					**Per 1 roll:**				
	Amount	Energy (kcal)	Protein (g)	Carbo-hydrate (g)	Fat (g)	Amount	Energy (kcal)	Protein (g)	Carbo-hydrate (g)	Fat (g)
flour*	20 cups	8800	320	1840	0	20 g	73	2.7	15.3	0
yeast	71 g	231	29	29	5	592 mg	2	0.2	0.2	0
sucrose	313 g	1221	0	313	0	2608 mg	10	0.0	2.6	0
salt	28 g	0	0	0	0	233 mg	0	0	0	0
calcium propionate	14.6 g	0	0	0	0	122 mg	0	0	0	0
whole milk	5 cups	744	38	59	40	10 ml	6	0.3	0.5	0.3
butter	284 g	2036	2.4	0.2	230	2367 mg	17	0	0.0	1.9
water	2.5 cups	0	0	0	0	5 ml	0	0	0	0
riboflavin	1.28 g	0	0	0	0	11 mg	0	0	0	0
						**total, 1 roll:**	109	3	19	2
						**total, 3 rolls:**	326	10	56	7

Supplementary Table 3

Choline was measured by MS at basal and 24 hours post bolus. Values are mean ± SEM. Metabolites were measured by MS at 24 hours post bolus (2.2 µmol d_9-choline). Data for the single perimenopausal female are include in All but not listed separately. Men n=23, Menopausal n=13, premenopausal n=38 and perimenopausal n=1.

	CHOLINE DIET 25%	CHOLINE DIET 50%	CHOLINE DIET 100%
**d_0-choline (µm)**			
**MEN**	10.1 ± 1.58	11.3 ± 2.04	12.6 ± 2.67
**PREMENOPAUSAL (F)**	8.74 ± 1.38	9.91 ± 2.06	10.7 ± 1.42
**MENOPAUSAL (F)**	10.7 ± 1.57	11.6 ± 2.54	13.3 ± 2
**ALL**	9.5 ± 1.7	11 ± 2.2	12 ± 2.3
**d_9-choline (µm)**			
**MEN**	0.02 ± 0.046	0.04± 0.047	0.04 ± 0.047
**PREMENOPAUSAL (W)**	0.00 ± 0.001	0.00 ± 0.045	0.00 ± 0.042
**MENOPAUSAL (W)**	0.00 ± 0.046	0.02 ± 0.052	0.03 ± 0.038
**ALL**	0.00 ± 0.046	0.02 ± 0.048	0.00 ± 0.046
**IER-choline (µm)**			
**MEN**	0.00 ± 0.004	0.00± 0.004	0.00 ± 0.003
**PREMENOPAUSAL (W)**	0.00 ± 0.005	0.00 ± 0.004	0.00 ± 0.004
**MENOPAUSAL (W)**	0.00 ± 0.004	0.00 ± 0.005	0.00 ± 0.030
**ALL**	0.00 ± 0.005	0.00 ± 0.004	0.00 ± 0.004

Supplementary Table 4

Linear mixed-effects model estimates (β coefficients and 95% confidence intervals) comparing circulating tHcy and liver fat (CAP) across dietary choline intake levels. The 100% Adequate Intake (AI) condition was used as the reference group. Fixed effects included choline intake and sex/hormonal status, while participant ID was included as a random intercept to account for repeated measures. Total homocysteine (tHcy) was significantly elevated in the 25% AI group. CAP measurements at Day 15 and change in CAP (diffCAP) trended higher with lower choline intake, although these differences were not statistically significant.

The only perimenopausal woman was not taking into account for this analysis.

Outcome	Comparisons	Beta (LCL, UCL)	*p*
**t HCY**	**ref: 100%AI**		
	**25% AI**	0.23 (0.01,0.45)	$4.47\text{x}10^{-2}$
	**50%AI**	-0.06 (-0.29 ,0.16)	$5.72\ \text{x}10^{-1}$
**CAP (d15)**	**ref: 100%AI**		
	**25% AI**	4.66 (-3.73,13.05)	$2.78\text{x}10^{-2}$
	**50%AI**	1.03 (-7.38, 9.45)	$8.10\text{x}10^{-1}$
**diffCAP**	**ref: 100%AI**		
	**25% AI**	-1.70(-12.92,9.43)	$7.65\text{x}10^{-1}$
	**50%AI**	-10.37(-21.49,0.77)	$7\text{x}10^{-2}$

Supplementary Table 5

Linear mixed-effects model estimates (β coefficients and 95% confidence intervals) for diet sequence (arm order) effects across metabolites and study outcomes. No significant effects of diet sequence were detected for any outcome (all p>0.05) confirming the absence of carry-over effects between dietary arms.

**Metabolite**		**Beta(LCL,UCL)**	***p***
Choline	ref:Arm 0.25_0.5_1		
	Arm0.25_1_0.5	0.05(-0.88,0.98)	0.921
	Arm0.5_0.25_1	0.43(-0.49,1.35)	0.389
	Arm0.5_1_0.25	-0.07(-1.04,0.91)	0.902
	Arm1_0.25_0.5	0.04(-0.82,0.89)	0.934
	Arm1_0.5_0.25	0.06(-1.03,1.16)	0.916
Betaine	ref:Arm 0.25_0.5_1		
	Arm0.25_1_0.5	-4.50(-14.96,5.96)	0.429
	Arm0.5_0.25_1	-0.80(-11.20,9.60)	0.887
	Arm0.5_1_0.25	0.15(-10.88,11.19)	0.979
	Arm1_0.25_0.5	-1.90(-11.59,7.78)	0.718
	Arm1_0.5_0.25	-0.87(-13.25,11.52)	0.897
PtdCho	ref:Arm 0.25_0.5_1		
	Arm0.25_1_0.5	358.99(-12.44,732.46)	0.079
	Arm0.5_0.25_1	109.94(-258.98,478.75)	0.583
	Arm0.5_1_0.25	150.91(-240.76,542.58)	0.479
	Arm1_0.25_0.5	-161.52(-506.46,182.44)	0.389
	Arm1_0.5_0.25	-34.03(-473.53,405.38)	0.886

Supplementary Table 5 (continued)

**Outcome**		**Beta(LCL,UCL)**	***p***
t HCY	ref:Arm 0.25_0.5_1		
	Arm0.25_1_0.5	0.18(-0.88,1.24)	0.753
	Arm0.5_0.25_1	0.15(-0.90,1.21)	0.790
	Arm0.5_1_0.25	-0.03(-1.15,1.09)	0.960
	Arm1_0.25_0.5	0.22(-0.77,1.20)	0.684
	Arm1_0.5_0.25	-0.81(-2.07,0.44)	0.236
CAP (D15)	ref:Arm 0.25_0.5_1		
	Arm0.25_1_0.5	11.99(-9.80,33.71)	0.312
	Arm0.5_0.25_1	13.29(-8.16,34.73)	0.257
	Arm0.5_1_0.25	14.89(-8.70,38.49)	0.248
	Arm1_0.25_0.5	1.27(-19.05,21.59)	0.908
	Arm1_0.5_0.25	16.32(-9.22,41.87)	0.243
diffCAP	ref:Arm 0.25_0.5_1		
	Arm0.25_1_0.5	-8.24(-24.84,8.65)	0.366
	Arm0.5_0.25_1	3.27(-12.80,19.48)	0.710
	Arm0.5_1_0.25	-3.56(-21.44,14.32)	0.710
	Arm1_0.25_0.5	-2.14(-17.50,13.47)	0.799
	Arm1_0.5_0.25	-1.57(-20.98,17.96)	0.882

Supplementary Figure 1. Plasma phosphatidylcholine and total homocysteine concentrations.

Plasma phosphatidylcholine (PtdCho) concentrations (A–D) and plasma total homocysteine (tHcy) concentrations (E–H) are shown at the end of each dietary arm (25%, 50%, and 100% of the Adequate Intake (AI) for choline). Graphs are stratified by sex and menopausal status: men, premenopausal women, menopausal women, and all participants. Each dot represents one participant; bars indicate group mean ± SEM.

Data were analyzed using a linear mixed-effects model with diet as a fixed effect and subject as a random effect, with Geisser-Greenhouse correction.

Post hoc comparisons were performed using Tukey's test, and reported p-values are adjusted for multiple comparisons.

Sample sizes: PtdCho- men, n = 23; premenopausal women, n = 38; menopausal women, n = 13. For the 50% AI arm, plasma samples could not be obtained for one man and one menopausal woman. The perimenopausal participant is included in "all participants'

Abbreviations: AI, Adequate Intake; PtdCho, phosphatidylcholine; tHcy, total homocysteine.

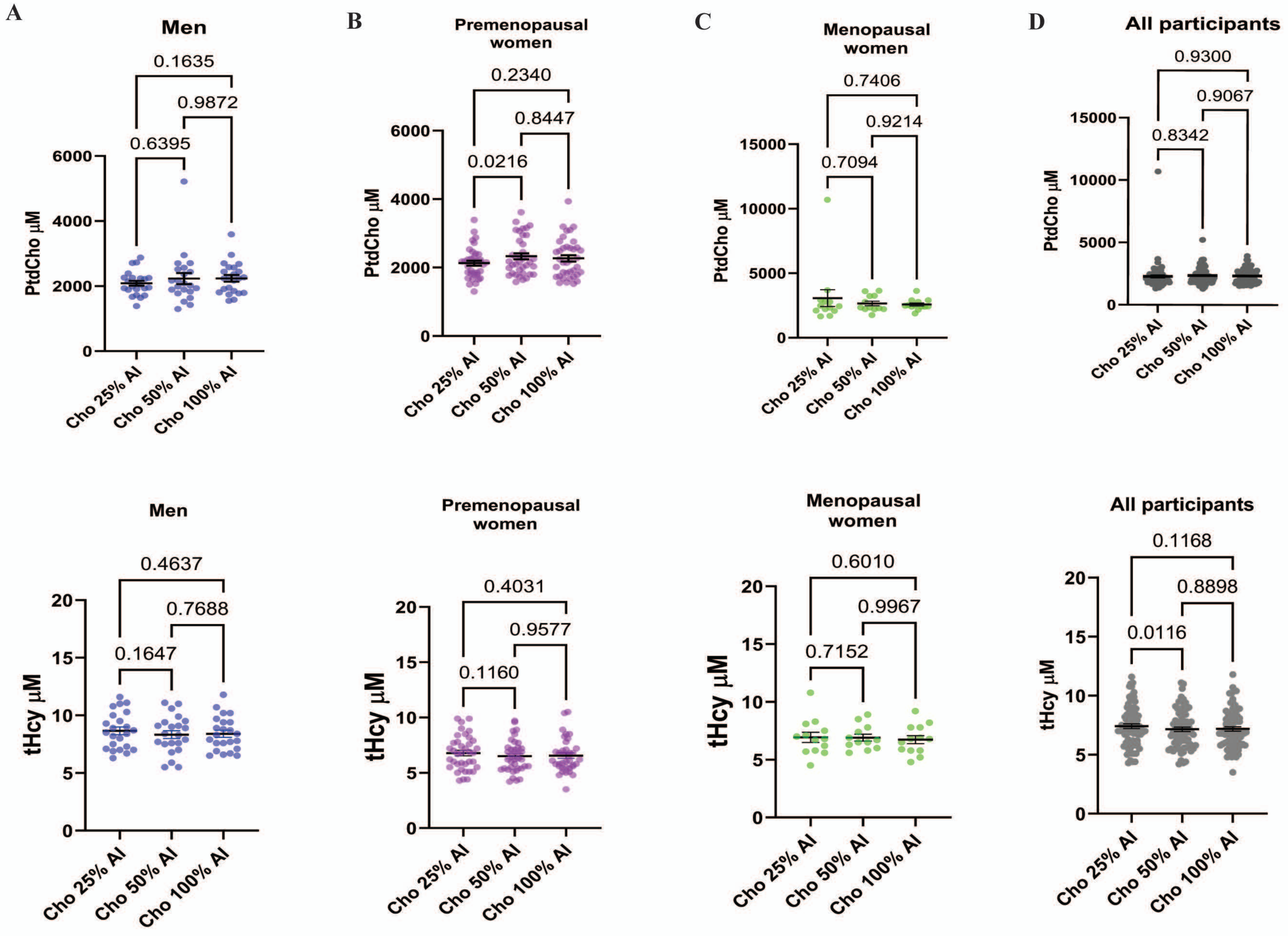

Supplementary Figure 2.

Aspartate aminotransferase (AST) and Alanine transaminase (ALT) concentrations across dietary choline intake stratified by sex and menopausal status. (A-D) AST levels (U/L) measured at the end of each choline intake condition (25%, 50% and 100% of AI, are shown for men (A), premenopausal women (B), menopausal women (C), and all participants (D). (E-H) ALT levels (U/L) at the same choline dietary conditions mentioned above, men (E), premenopausal women (F), menopausal women (G) and all participants (H). Each dot represents one participant; bars represent the group mean ± SEM. Data were analyzed using a linear mixed-effects model with a fixed effect for diet and a random effect with Geisser-Greenhouse correction.
Post hoc comparisons were performed using Tukey's test and reported p values are adjusted for multiple comparisons. Sample sizes: men, n = 23; premenopausal women, n = 37; menopausal women, n = 14. For the 50% AI arm, plasma samples could not be obtained for one man and one menopausal woman.
Abbreviations: AI, Adequate Intake; AST, aspartate aminotransferase; ALT, alanine transaminase.

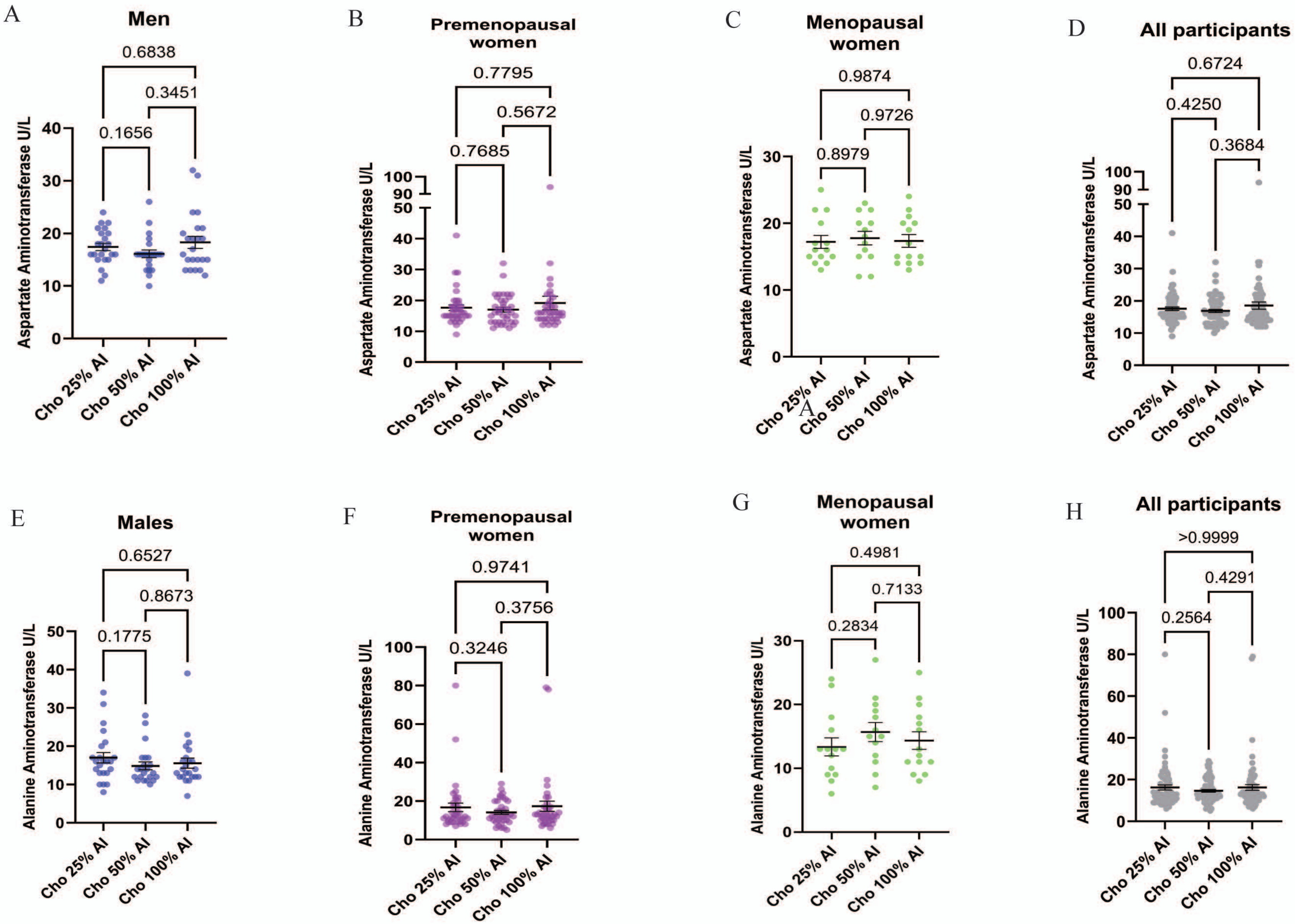

Supplementary Figure 3.

CAP (controlled attenuation parameter) measured by Fibroscan at baseline and at the end of each dietary choline intake condition.

(A-D) CAP values (dB/m) on Day1 prior to diet exposure are shown for men (A), premenopausal women (B), menopausal women (C) and all participants (D). Each dot represents on participant, and horizontal bars indicate group means. Data were analyzed using a linear mixed-effects model with Geisser-Greenhouse correction. Post hoc comparisons were performed using Tukey's test, and reported p-values are adjusted for multiple comparisons.

(E) Individual slope graphs showing CAP values at the end of the 100%, 50% and 25% AI dietary choline intake conditions. Each line represents one participant.

(F) Individual slope graphs showing changes in CAP measured by Fibroscan at the end of 100% AI and 25% AI dietary choline intake.

Each line represents one participant, with CAP values (dB/m) measure at the end of each condition. These participants did not show a 10% increase in liver fat when consuming 25% AI choline.

Sample sizes men n=22 (25% AI and 100% AI) and n=20 (50% AI), premenopausal women n=33 (25% AI) 36 (50% AI) and 35 (100% AI), menopausal women n=14 (25% AI and 100% AI) and n=13 (50% AI). For the Fibroscan, some data files could not be retrieved from the equipment. Analyses were conducted under the assumption that this data loss was random.

Abbreviations: AI, Adequate Intake, CAP, controlled attenuation parameter.

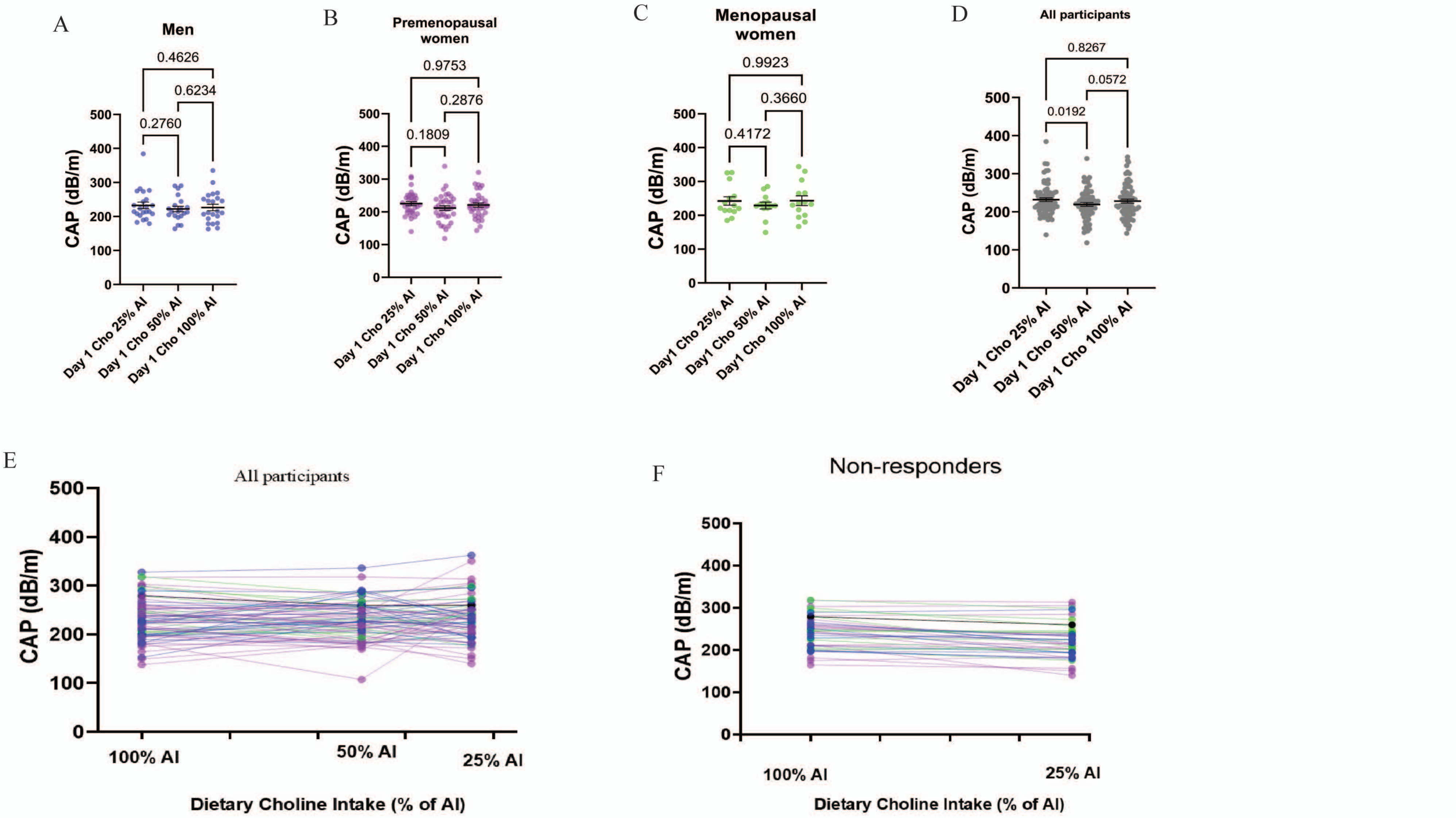

Supplement: 1 [file NIHMS2154307-supplement-1.pdf]
